# Percutaneous Transluminal Angioscopy During Coronary Intervention

**DOI:** 10.1155/DTE.7.15

**Published:** 2000

**Authors:** Kyoichi Mizuno, Shunnta Sakai, Shinnya Yokoyama, Takayoshi Ohba, Ryuta Uemura, Yasutsugu Seimiya, Masamitsu Takano, Jun Tanabe, Masato Tomimura, Takahiro Imaizumi, Shu Mei Ma, Shigenobu Inami, Kentarou Okamatsu, Noritake Hata

**Affiliations:** 1 Cardiovascular Center and Department of Internal Medicine Chiba Hokusoh Hospital Nippon Medical School 1715 Kamagari, Inba Chiba 270-1694 Japan

## Abstract

To investigate the feasibility of angioscopic-guided percutaneous transluminal coronary angioplasty and to elucidate the mechanism of efficacy of coronary stenting for acute myocardial infarction, we performed coronary angioscopy in 102 patients with stable angina or acute myocardial infarction. Thrombi and intimal flaps were observed in most patients after coronary angioplasty. Large intimal splits were seen in one third of patients. Stents were inserted in 10 patients who were revealed to have a large flap or protruding split to the inner lumen. Thrombolytic agents were administered in 2 patients with large thrombi. Additional treatments were required in 32% of patients. No acute myocardial infarction or unstable angina occurred in patients during hospitalization. Thus, angioscopy of the coronary lumen enables clinicians to determine the most appropriate and least risky coronary intervention strategy. In patients with acute myocardial infarction, angioscopy revealed occlusive or protruding thrombi in 34 of 35 patients. The protruding thrombi disappeared after stenting. The frequency of large intimal flaps increased after predilatation with balloon, but these disappeared after stenting. The present angioscopic study demonstrates that the coronary stent compresses the occlusive or protruding thrombi and covers the ruptured thrombogenic plaque Consequently, smooth-surfaced and wide vessel lumen are obtained.


*Diagnostic and Therapeutic Endoscopy*, Vol. 7, pp. 15–20
Reprints available directly from the publisher
Photocopying permitted by license only

© 2000 OPA (Overseas Publishers Association) N.V.
Published by license under
the Harwood Academic Publishers imprint,
part of The Gordon and Breach Publishing Group.
Printed in Malaysia.

### Percutaneous Transluminal Angioscopy during Coronary Intervention

KYOICHI MIZUNO*, SHUNNTA SAKAI, SHINNYA YOKOYAMA, TAKAYOSHI OHBA, RYUTA UEMURA, YASUTSUGU SEIMIYA, MASAMITSU TAKANO, JUN TANABE, MASATO TOMIMURA, TAKAHIRO IMAIZUMI, SHU MEI MA, SHIGENOBU INAMI, KENTAROU OKAMATSU and NORITAKE HATA

*Cardiovascular Center and Department of Internal Medicine, Chiba Hokusoh Hospital, Nippon Medical School, 1715 Kamagari, Inba, Chiba, 270-1694, Japan*

*(Received 2 May 2000; Revised 13 June 2000; In final form 19 June 2000)*

**To investigate the feasibility of angioscopic-guided percutaneous transluminal coronary angioplasty and to elucidate the mechanism of efficacy of coronary stenting for acute myocardial infarction, we performed coronary angioscopy in 102 patients with stable angina or acute myocardial infarction. Thrombi and intimal flaps were observed in most patients after coronary angioplasty. Large intimal splits were seen in one third of patients. Stents were inserted in 10 patients who were revealed to have a large flap or protruding split to the inner lumen. Thrombolytic agents were administered in 2 patients with large thrombi. Additional treatments were required in 32% of patients. No acute myocardial infarction or unstable angina occurred in patients during hospitalization. Thus, angioscopy of the coronary lumen enables clinicians to determine the most appropriate and least risky coronary intervention strategy. In patients with acute myocardial infarction, angioscopy revealed occlusive or protruding thrombi in 34 of 35 patients. The protruding thrombi disappeared after stenting. The frequency of large intimal flaps increased after predilatation with balloon, but these disappeared after stenting. The present angioscopic study demonstrates that the coronary stent compresses the occlusive or protruding thrombi and covers the ruptured thrombogenic plaque. Consequently, smooth-surfaced and wide vessel lumen are obtained.**

*Keywords*: PTCA, Acute myocardial infarction, Stable angina, Coronary stent

### INTRODUCTION

Percutaneous transluminal coronary angioscopy is a new diagnostic tool that permits non-operative imaging of intravascular structures. It provides a precise, full-color, three-dimensional perspective of the interior surface of coronary arteries [1,2]. The high resolution of images can disclose luminal changes in minute plaque rupture, ulceration, intimal flap or torn tissue strands not typically appreciated by coronary arteriography [3,4]. Color discrimination makes it relatively easy to

* Corresponding author. Tel.: +81-476-99-1111. Fax: +81-476-99-1863. E-mail: mizunok@nms.ac.jp.

distinguish between a thrombus and a plaque [5,6]. Therefore, angioscopy facilitates not only in the correlation of anatomical and pathological features but also in the monitoring of coronary interventions. Recently, the coronary stent has been widely used for the management of abrupt or threaded occlusion during percutaneous transluminal coronary angioplasty (PTCA) (bailout) and for the management of acute myocardial infarction [7]. However, why stenting is efficacious against acute myocardial infarction remains unclear. The purpose of this study is to investigate the feasibility of angioscopic-guided PTCA and to elucidate the mechanism of efficacy of coronary stenting for acute myocardial infarction.

### CORONARY ANGIOSCOPY

Coronary angioscopy was performed using of a 4.5 F monorail typed rapid exchange angioscope. Before coronary angioscopy, the white balance was adjusted for color correction. The coronary lumen could been observed in 5 cm long segments by inflating the occlusion cuff on the outer catheter and by moving the optic bundle. Warm saline (0.6–0.8 ml/sec) was injected into the coronary lumen to obtain a clear view. Light power was adjusted to avoid refraction and to obtain adequate color. The images were displayed on the monitor and recorded on S-VHS. Angioscopic findings can be classified into the following 7 categories: thrombus, hemorrhage, dissection, intimal flap, intimal split, ulceration and stable atheroma, according to color, mobility, irregularity of intraluminal surface, shape and protrusion into the inner lumen.

### ANGIOSCOPIC-GUIDED PTCA

### Patients

Forty patients diagnosed with stable angina or old myocardial infarction underwent coronary angioscopy immediately after PTCA. PTCA was successfully in all patients by angiographic criteria (residual minimum lumen diameter $\leqq 50\%$).

### Results

Immediately following coronary intervention, the angioscopic visualization of 40 lesions of the 40 patients were reviewed in the cardiac cathetelization room. Angioscopy could not be reviewed in 3 patients because of delivery failure of the angioscope (2 patients) and inadequate visualization (1 patient). Therefore, 37 patients comprised the study population. The baseline clinical and angiographic characteristics of these 37 patients are shown in Table I. Thrombi were observed in most patients after angioplasty despite the use of anticoagulant and antiplatelet agents before and the during procedure. Intimal flaps were also observed in most patients. Large intimal splits were seen in one third of patients (Table II). Angiography

TABLE I Angioscopic findings immediately after PTCA in 37 patients

| | |
|---|---|
| Thrombus | 35 (96%) |
| Hemorrhage | 3 (80%) |
| Intimal flap | 34 (92%) |
| Intimal split | 12 (32%) |

TABLE II Baseline characteristics of patients with stable angina ($n=37$) who underwent angioscopic-guided PTCA

| | |
|---|---|
| Age (mean ± SD) | 61 ± 9 |
| Men | 27 (73%) |
| Hypertension | 23 (62%) |
| Diabetes millitus | 9 (24%) |
| Hyperlipidemia | 21 (57%) |
| Smoker | 14 (37%) |
| Previous MI | 11 (30%) |
| *Target vessel* | |
| LAD | 21 (57%) |
| RCA | 12 (32%) |
| Lcx | 4 (11%) |
| *No. of diseased vessels* | |
| 1 vessel | 21 (57%) |
| 2 vessels | 8 (22%) |
| 3 vessels | 8 (22%) |

MI: Myocardial infarction, LAD: Left anterior descending, RCA: Right coronary artery, Lcx: Left circumflex, No.: Number.

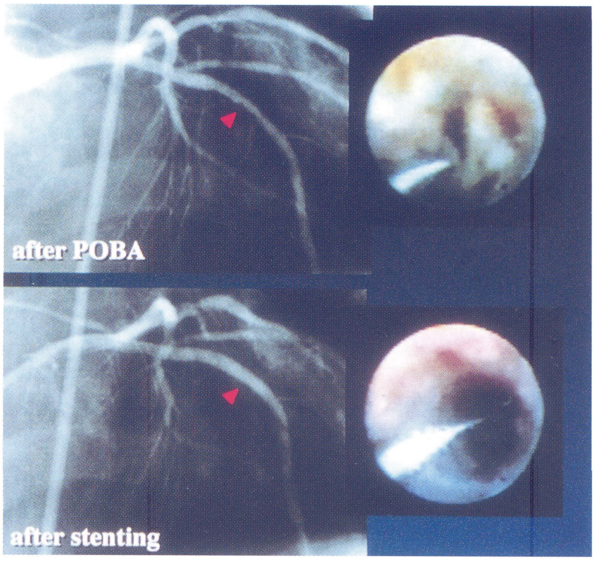

FIGURE 1 Angioscopic-guided PTCA. After balloon angioplasty (POBA), large protruding disruption was observed by angioscopy (right upper), but coronary arteriography failed to disclose disruption (left upper). After stenting, disruption was sealed with stent (right lower). Large coronary lumen was obtained (left lower).

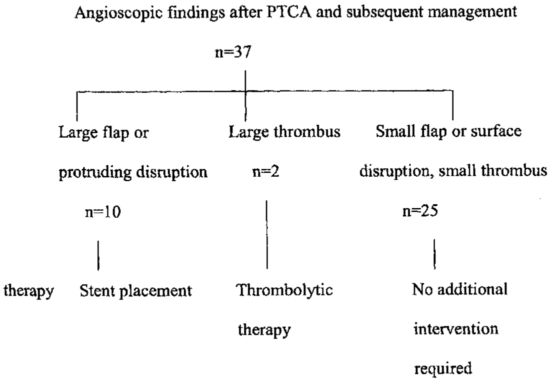

FIGURE 2 Changes in management due to angioscopy during PTCA. Angioscopy influenced clinical management in 12 of 37 (32.4%) patients.

revealed intimal flaps in only 2 patients and thrombi in 1 patient. A stent was inserted in 10 patients who had a large flap or protruding split to the inner lumen in order to prevent abrupt occlusion of the coronary artery. After the insertion of the stent, large intimal flaps or protruding disruptions were sealed with the stent (Fig. 1). Only tiny flaps were revealed by angioscopy. After stenting, no acute or subacute coronary occlusions occurred. Tissue plasminogen activator was administered intracoronary using the guide catheter in 2 patients with large thrombi. Thrombi were partially dissolved. Additional treatment during coronary intervention was provided in 12 of 37 patients (32.4%) (Fig. 2). No acute myocardial infarction or unstable angina was observed in patients during hospitalization, nor at 6-month postoperative follow-up.

### THE MECHANISM OF EFFICACY OF STENT AGAINST ACUTE MYOCARDIAL INFARCTION

### Patients

Primary stenting was performed in 65 patients diagnosed with acute myocardial infarction. Thirty-five of these patients had undergone successful coronary angioscopy before coronary intervention by predilatation with a balloon and stenting. Baseline clinical and angiographic characteristics of the patients are shown in Table III.

TABLE III Baseline characteristics of primary stenting in patients with acute myocardial infarction ($n = 35$)

| | |
|---|---|
| Age (mean ± SD) | 60 ± 9 |
| Men | 27 (75%) |
| Hypertension | 18 (51%) |
| Diabetes millitus | 10 (29%) |
| Hyperlipidemia | 22 (63%) |
| Smoker | 22 (63%) |
| Previous MI | 6 (17%) |
| Infarct location | |
| Anterior | 19 (54%) |
| Inferior | 13 (37%) |
| Portal ateral | 3 (9%) |
| *Target vessel* | |
| LAD | 17 (49%) |
| RCA | 13 (37%) |
| Lcx | 5 (14%) |
| *No. of diseased vessels* | |
| 1 vessel | 20 (57%) |
| 2 vessels | 11 (31%) |
| 3 vessels | 4 (12%) |

MI: Myocardial infarction, LAD: Left anterior descending, RCA: Right coronary artery, Lcx: Left circumflex, No.: Number.

### Results

Thrombi were observed in 34 of 35 patients (97%). All thrombi were occlusive or protruding before coronary intervention. After predilatation with a balloon, the frequency of protruding occlusive thrombi decreased from 97% to 51% (16 of 35). Mural thrombi were observed in the remaining patients. After stenting, protruding thrombi disappeared in all patients (Fig. 3). Large intimal flaps were observed in 11 of 35 patients (31%) before intervention. The frequency of large intimal flaps increased after predilatation with a balloon from 31% to 97%. However, large intimal flaps were disappeared after stenting in all patients (Fig. 4).

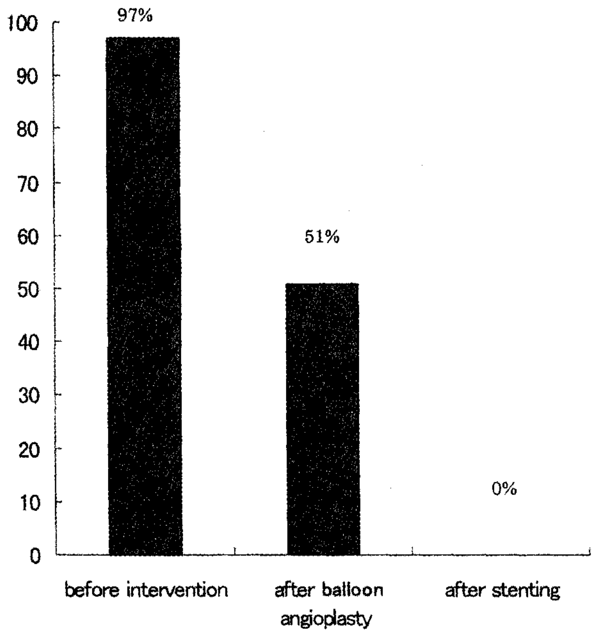

FIGURE 3 Changes in protruding thrombi before and after stenting in patients with acute myocardial infarction.

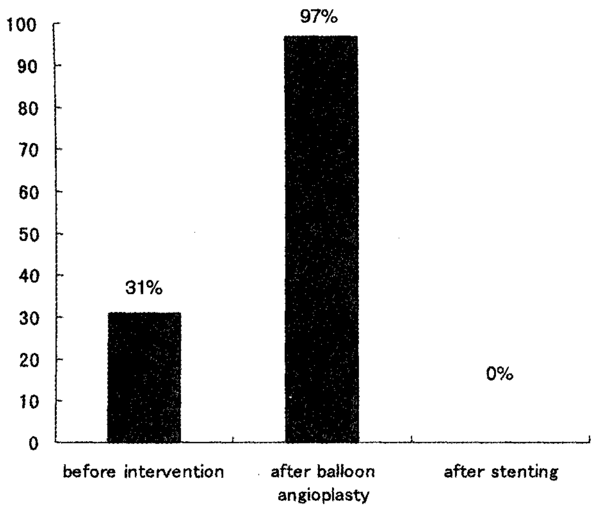

FIGURE 4 Changes in large flaps before and after stenting in patients with acute myocardial infarction.

### DISCUSSION

### Angioscopic-guided PTCA

Clinicians have conventionally used angiography to determine the clinical outcome of coronary intervention. Unfortunately, angiographic images capture only luminal features and, as such, do not accurately assess most endovascular therapies. Although intravascular ultrasoundography provides cross-sectional images useful in the assessment and guidance of coronary interventions [8], intravascular ultrasound images are limited in specificity for thrombus formation and intimal flaps. In the present study, angioscopy indicated that intimal flaps and thrombi were often present after balloon angioplasty. Previously we reported that the presence of a large flap, detected by angioscopy, was associated with acute coronary occlusion after conventional PTCA [9]. Other investigators [10,11] using angioscopy also showed that the primary cause of post-angioplasty occlusion was intimal flap in the majority of cases, in contrast to a thrombus in only a few cases. Although previous angioscopic research identified the cause of acute occlusion after PTCA, few reports have reported the efficacy of angioscopic guidance in optimal coronary interventions. We inserted the stent at the site of the large flap or protruding disruptions which were observed by angioscopy, and thrombolytic therapy was provided for patients with large thrombi after PTCA. No recurrent ischemia occurred during patients' hospital stay following completion of this therapy.

Our present results support the findings of Teirstein et al. [12] by confirming the effectiveness of angioscopy during coronary stenting. Clinical decisions directly influenced by angioscopy in Teirstein et al.'s study included the initiation of intracoronary thrombolytic therapy for a thrombus visualized angioscopically, repeat angioplasty when forming plaque was seen to be bulging into the lumen at the stent articulation site, and the replacement of additional stents replaced when angioscopy revealed significant proximal or distal disease/ or an unsuspected gap between 2 tandem stents.Angioscopy influenced the clinical management of 18 (37.5%) of their patients. Likewise, Mirecki et al. [13] reported that angioscopy changed clinical management in 75% of patients, obviating the need for thrombolytic therapy and mechanical intervention, and altering the mechanical intervention chosen. In the present study, angioscopy influenced the clinical management of 12 out of 37 (33%) patients. Thus, angioscopy of the coronary lumen enables clinicians to determine the most appropriate and least risky coronary intervention strategy for a given patient. Furthermore, angioscopy was useful for the prediction and the prevention of acute occlusion after PTCA.

### THE MECHANISM OF FEASIBILITY OF STENT FOR ACUTE MYOCARDIAL INFARCTION

Early in the implantation of stents, it was thought that the use of these may be contraindicated if a thrombus was present in the infarcted vessel [14,15]. Recently, however, many reports have shown that coronary stenting is feasible after acute myocardial infarction and is actually associated with excellent short-term outcomes [7]. Interestingly, this may turn out to be one of the most important applications of stenting despite the early concerns about stent thrombosis. However, the mechanism of favourable outcomes remains to be elucidated. We used angioscopy to examine the morphological characteristics of the infarction-related lesion before and after stenting. Smoothly wide lumina without large intimal flaps or multiple lining thrombi but not occlusive or protrusive thrombi were observed after stenting. The present angioscopic study demonstrates that coronary stents compress the occlusive or protruding thrombi and cover the ruptured thrombogenic plaques. Consequently, smooth-surfaced wide vessel lumen are obtained. These findings reveal that the utilization of stenting as an acute-stage intervention in patients with acute myocardial infarction induces more favorable clinical outcomes.

### *References*

[1] Mizuno, K., Arai, T., Satomura, K. *et al.* New percutaneous transluminal coronary angioscope. *J. Am. Coll. Cardiol.* 1989; **13**: 363–368.

[2] Sherman, C.T., Litvack, F. and Grundfest, W. *et al.* Coronary angioscopy in patients with unstable angina pectoris. *N. Engl. J. Med.* 1986; **315**: 913–919.

[3] Mizuno, K., Miyamoto, A., Satomura, K. *et al.* Angioscopic coronary macromorphology in patients with acute coronary disorders. *Lancet* 1991; **337**: 809–812.

[4] Ramee, S.R., White, C.J., Collins, T.J., *et al.* Percutaneous angioscopy during coronary angioplasty using a steerable microangioscope. *J. Am. Coll. Cardiol.* 1991; **17**: 100–105.

[5] Uchida, Y., Hasegawa, K., Kawamura, K. *et al.* Angioscopic observation of the coronary luminal changes induced by percutaneous transluminal coronary angioplasty. *Am. Heart* 1989; **117**: 769–776.

[6] Mizuno, K., Satomura, K., Miyamoto, A. *et al.* Angioscopic evaluation of coronary-artery thrombi in acute coronary syndromes. *N. Engl. J. Med.* 1992; **326**: 287–291.

[7] Gregg, W.S. Prospective, multicenter study of the safety and feasibility of primary stenting in acute myocardial infarction: in-hospital and 30-day result of the PAMI stent pilot trial. *J. Am. Cardiol.* 1998; **31**: 23–30.

[8] Colombo, A., Hall, P., Nakamura, S. *et al.* Intracoronary stenting without anticoagulation accomplished with intravascular ultrasound guidance. *Circulation* 1995; **91**: 1676.

[9] Mizuno, K., Ohkuni, S., Takano, M. *et al.* Usefulness of coronary angioscopy during coronary intervention. *J. Jpn. Coll. Angiol.* 1999; **39**: 33.

[10] White, C.J., Ramee, S.R., Collins, T.J. *et al.* Coronary angioscopy of abrupt occlusion after angioplasty. *J. Am. Coll. Cardiol.* 1995; **25**: 1681–1684.

[11] Waxman, S., Sassower, M.A., Mittleman, M.A. *et al.* Angioscopic predictors of early adverse outcome after coronary angioplasty in patients with unstable angina and non-Q wave myocardioal infarction. *Circulation* 1996; **93**: 2106–2113.

[12] Teirstein, P.S., Schatz, R.A., Chui Wong, S. *et al.* Coronary stenting with angioscopic guidance. *Am. J. Cardiol.* 1995; **75**: 344–347.

[13] Mirecki, F., Sharaf, B., Williams, D. Intracoronary angioscopy impacts clinical decision making in patients with acute coronary syndromes. *J. Am. Coll. Cardiol.* 1994; Abst: 170A.

[14] Monassir, J.P., Hamon, M., Elias, J. *et al.* Early versus late coronary stenting following acute myocardial infarction: Result of the STENTIM I study (French registry of stenting in acute myocardial infarction). *Cathet Cardiovasc Diagn* 1997; **42**: 243–248.

[15] Urban, P., Macaya, C., Rupprecht, H.J. *et al.* For the MATTIS Investigators. Randomized evaluation of anticoagulation versus antiplatelet therapy after coronary stent implantation in high risk patients. The Multicenter Aspirin and Ticlopidine Trial after Intracoronary Stenting (MATTIS). *Circulation* 1998; **98**: 2126–2132.

